# Transcriptomics and Metabolomics Changes Triggered by Inflorescence Removal in *Panax notoginseng* (Burk.)

**DOI:** 10.3389/fpls.2021.761821

**Published:** 2021-11-12

**Authors:** Yu Bai, Haijiao Liu, Jianying Pan, Shiyan Zhang, Yixin Guo, Yisha Xian, Zhirong Sun, Zilong Zhang

**Affiliations:** School of Chinese Materia Medica, Beijing University of Chinese Medicine, Beijing, China

**Keywords:** *Panax notoginseng*, inflorescence removal, transcriptomics, metabolomics, saponins, flavonoid

## Abstract

The root of *Panax notoginseng* (Burk.), in which saponins are the major active components, is a famous traditional Chinese medicine used to stop bleeding and to decrease inflammation and heart disease. Inflorescence removal increases the yield and quality of *P. notoginseng*, but the underlying molecular mechanisms are unknown. Here, the differences between inflorescence-removal treatment and control groups of *P. notoginseng* were compared using transcriptomics and metabolomics analyses. Illumina sequencing of cDNA libraries prepared from the rhizomes, leaves and roots of the two groups independently identified 6,464, 4,584, and 7,220 differentially expressed genes (DEG), respectively. In total, 345 differentially expressed transcription factors (TFs), including MYB and WRKY family members, were induced by the inflorescence-removal treatment. Additionally, 215 DEGs involved in saponin terpenoid backbone biosynthetic pathways were identified. Most genes involved in the mevalonic acid (MVA) and methylerythritol phosphate (MEP) pathways were activated by inflorescence removal. The co-expression analysis showed that the low expression levels of flavonoid biosynthesis-related genes (e.g., *C4H* and *F3H*) decreased the biosynthesis and accumulation of some flavonoids after inflorescence removal. The results not only provide new insights into the fundamental mechanisms underlying the poorly studied inflorescence-removal process in *P. notoginseng* and other rhizome crops, but they also represent an important resource for future research on gene functions during inflorescence-removal treatments and the reproductive stage.

## Introduction

*Panax notoginseng* (Burk.) F.H. Chen is a perennial herb that belongs to the Araliaceae family of the genus Panax (Li et al., 2009). *P. notoginseng* is an important traditional Chinese herbal medicine that is mainly grown in Yunnan Province, China. It has been cultivated and used medicinally since ancient times because of its remarkable and valuable hemostatic effects (Briskin, 2000). The main active ingredients of *P. notoginseng* are *P. notoginseng* saponins (PNSs), which have diverse biological activities, such as membrane-permeabilizing, immunostimulating, hypocholesterolemic, anti-carcinogenic and anti-microbial (Ng, 2006). Because of its numerous health benefits, *P. notoginseng* is the main raw material of several Chinese patent medicines, such as Yunnan baiyao, Pien Tze Huang, Xuesaitong and Sanqi Tongshu Capsules. Thus, *P. notoginseng* is of high medicinal and economic value.

During the production process, farmers often remove the flower buds of *P. notoginseng* to increase its yield. In addition, removing the flower buds in the third year significantly increases the saponins content in *P. notoginseng* roots (Xia et al., 2017). However, the molecular mechanisms by which flower removal increases the yield and quality of *P. notoginseng* are still unclear. Moreover, limited studies have focused on the molecular regulation of flower removal or reproductive growth on root secondary metabolism.

*Panax notoginseng* saponins are thought to derive from pathways that lead to the synthesis of isoprenoids. These precursors of PNS are synthesized in the mevalonic acid (MVA) pathway in the cytosol and the methylerythritol phosphate (MEP) pathway in the plastid, resulting in the synthesis of 2,3-oxidosqualene (Luo et al., 2011). The subsequent modification of 2,3- oxidosqualene by multiple oxidations, which are mediated by cytochrome P450-dependent monooxygenases, and glycosylations results in a wide variety of PNSs. Many transcripts related to saponin synthesis have been selected using transcriptome analyses (Augustin et al., 2011; Wang et al., 2012; Kim et al., 2015). Recently, a large-scale transcriptome analysis of *P. notoginseng* identified 270 unigenes putatively involved in triterpene saponin biosynthesis, as well as cytochrome P450 and glycosyltransferase -like genes that may be involved in the conversion of the triterpene saponin backbone into different PNSs (Liu et al., 2015). The protopanaxadiol (PPD)- and protopanaxatriol-type saponin contents in *P. notoginseng* are also significantly correlated with CYP716A47 and CYP716A53v2 expression levels, respectively (Augustin et al., 2011). Thus, we hypothesized that flower removal changed the expression patterns of transcripts participating in saponin synthesis in *P. notoginseng* root tissues.

In addition to saponins, some flavonoids are also active components of *P. notoginseng*. Flavonoids, which belong to the phenolic class of compounds (Fang et al., 2017), include flavones, flavonoids, isoflavones, anthocyanins, flavanols, flavonols, and derivatives (e.g., catechins) (Gao et al., 2019). Most genes have been actively studied for their contributions to flavonoid accumulation in several plants, including Arabidopsis (*Arabidopsis thaliana*) (Kim et al., 2017), maize (*Zea mays*) (Jin et al., 2017), figs (*Ficus carica* L.) (Wang et al., 2017), and tea plant (*Camellia sinensis*) (Wu et al., 2019). The genes that regulate flavonoid biosynthesis, except for 4CL and F3′5′H, have higher transcription levels in red cotton than in white cotton (Long et al., 2019). Under the regulation of the *CsFLS* gene, the flavonoid content of tea made from the albino cultivar ‘Rougui’ is greater than that made from normal green-leaf cultivars (Wang et al., 2020). However, there is limited information on flavonoid levels in inflorescence removal-treated plants.

Owing to the development of bioinformatics tools and resources, it is efficient to analyze complex responses to external interventions by integrating multi-omics data (Masclaux-Daubresse et al., 2014). For example, metabolites and genes involved in responses to exogenous abscisic acid in tea plants have been identified through the integration of metabolomics and transcriptomics data (Gai et al., 2020). In addition, some candidate genes and metabolites in the adaptation of wheat (*Triticum aestivum*, L.) to Ultraviolet radiation have been identified by transcriptomics and metabolomics analyses (Wang et al., 2019). Here, the aim was to clarify the mechanisms underlying the effects of flower removal in *P. notoginseng* on saponin and flavonoid biosynthesis using transcriptomics integrated with metabolomics after an inflorescence-removal treatment. The hope is that the effects of flower removal will be explained from an integrative omics perspective and provide a theoretical basis and guidance for the growth of *P. notoginseng*.

## Materials and Methods

### Plant Materials

Three-year-old flowering-stage *P. notoginseng* plants, having similar growth vigor levels, were used in this study. The experiment was conducted in the production base of Wenshan in Yunnan Province (23°30′22.63″ N, 104°02′45.92″ E, altitude 1,400 m). The flower buds of the *P. notoginseng* plants were removed in early August. The leaves (L), taproots (R), and rhizomes (J) of *P. notoginseng* with and without removed buds were collected in October. In total, 15 plants were collected per group. The samples were carefully washed and stored for metabolomics and transcriptomics analyses.

### RNA Extraction, Illumina Library Construction and Sequencing

Total RNA was extracted using a Spin Column Plant Total RNA Purification Kit following the manufacturer’s protocol (Sangon Biotech, Shanghai, China). The samples included the leaves (L), taproots (R), and rhizomes (J) of *P. notoginseng* with buds removed (A) and *P. notoginseng* without buds removed (B) harvested in triplicate. The purity of the extracted RNA was assessed on 1% agarose gels and using a NanoPhotometer spectrophotometer (IMPLEN, Los Angeles, CA, United States). For RNA quantification, a Qubit RNA Assay Kit and a Qubit 2.0 Fluorometer (Life Technologies, Carlsbad, CA, United States) were used. Further, RNA integrity was assessed using an RNA Nano 6000 Assay Kit and the Agilent Bioanalyzer 2100 system (Agilent Technologies, Santa Clara, CA, United States). The construction of Illumina sequencing libraries was carried out as previously described (Chen et al., 2013). The cDNA libraries were sequenced on the Illumina HiSeq platform (Illumina Inc., San Diego, CA, United States) by Wuhan MetWare Biological Science and Technology Co., Ltd.^1^ (Wuhan, China).

### Sequencing Data Analysis

The clean reads were retrieved after trimming adapter sequences and removing low-quality reads with unknown nucleotides. A GC content distribution check was performed. To stitch clean reads, Trinity was used (Version r20140717). For hierarchical clustering, Corset was used^2^. The longest cluster sequence was obtained by clustering using Corset hierarchy as ‘Unigene’ for subsequent analysis. The assembled unigenes were then aligned with various databases, such as the Kyoto encyclopedia of genes and genomes (KEGG), non-redundant (NR), Swiss-Prot, Clusters of Orthologous Groups (COG), egNOG, euKaryotic Ortholog Groups (KOG), gene ontology (GO) and Pfam databases using a BLAST algorithm with a threshold of *E*-value < 1.0 × 10^–5^. The analogs of the unigene amino acid sequences were used as query against the Pfam database with the HMMER tool at a threshold of *E*-value < 0.01. The sequenced reads were compared with the unigene library using Bowtie, and the level of expression was estimated in combination with RSEM tool. The gene expression levels were determined in accordance with the FPKM values.

The read count was normalized and the DESeq2 was used to determine the differentially expressed genes (DEGs) between A and B, with the criteria | Log_2_Fold Change| ≥ 1 and false discovery rate (FDR) < 0.05. The GO enrichment analysis was performed using the topGO method based on the wallenius non-central hypergeometric distribution. The KEGG pathway enrichment analysis of the DEGs was performed using KOBAS2.0.

### Metabolome Analysis

Sample preparation, metabolite extraction and analysis were carried out as described previously (Shen et al., 2015). In brief, the 100-mg freeze-dried samples were extracted using 1.0 mL pure methanol (containing 0.1% formic acid). Subsequently, samples were centrifuged at 10,000 rpm for 10 min, and then, the extracts were absorbed and filtered. The extracted samples (2 μL) were analyzed using an HPLC system (Shimpack UFLC SHIMADZU CBM 30A) equipped with a Waters ACQUITY UPLC HSS T3 C18 column (1.8 μm, 2.1 mm × 100 mm). LIT and triple quadrupole scans were acquired on a QTRAP-MS equipped with an ESI Turbo Ion-Spray interface on an AB Sciex QTRAP 4,500 System in positive ion mode and controlled by Analyst 1.6.1 software. The solvent system, gradient program and ESI source operation parameters were as described previously (Chen et al., 2013).

The qualitative analyses of primary and secondary MS data were performed by searching the internal database using a self-compiled database of Wuhan MetWare Biological Science and Technology Co., Ltd. Data pre-processing and metabolite identification were performed using standard metabolic procedures. The variable importance of the projection (VIP) score of the application (O) PLS model was used to filter the best differentiated metabolites between treatments. Metabolites with significant differences in content were set with thresholds of VIP ≥ 1 and fold change ≥ 2 or ≤0.5. A Principal component analysis (PCA) was used to analyze the variability between groups and within groups. The functional annotation of differentially accumulated metabolites (DAMs) was performed based on KEGG pathways.

### Co-joint Analysis of the Transcriptome and Metabolome

The differential genes and metabolites were mapped onto the KEGG pathways at the same time. The enrichment results of the differential metabolites and genes were used to show the degree of pathway enrichment. To study the correlations between the genes and metabolites, the Corson program in *R* was used to calculate Pearson’s correlation coefficients, which were presented as a heat map.

## Results

### Overview of the *P. notoginseng* Transcriptomic Analysis

Illumina HiSeq paired-end sequencing technology was used to analyze the transcriptome of samples (L, R, and J) of *P. notoginseng*. In total, 18 libraries were constructed and sequenced. They represented three different organs from the treatments A and B, each in triplicate. A total of 981.3 million high-quality clean reads were obtained from the 18 libraries. The Q20 of all libraries was >97.86%, the Q30 was >93.57%, and the GC content was approximately 43% (Table 1). The *de novo* assembly of the clean reads of *P. notoginseng* resulted in 240,748 transcripts with an average contig size of 1,216 bp and an N50 contig size of 2,045 bp (Supplementary Table 1).

**TABLE 1:** Summary of the sequencing data from the 18 *P. notoginseng* samples.

Sample	Clean reads	Clean base (G)	Q20 (%)	Q30 (%)	GC content (%)
A-J-1	45570294	6.84	98.06	94.07	42.67
A-J-2	59617132	8.94	97.96	93.79	42.77
A-J-3	54803758	8.22	97.89	93.65	42.74
A-L-1	50931676	7.64	98.06	94.03	43.44
A-L-2	47157468	7.07	97.95	93.77	43.19
A-L-3	53220618	7.98	97.93	93.75	43.14
A-R-1	56873734	8.53	98.07	94.08	42.7
A-R-2	56412762	8.46	98.05	94.03	42.9
A-R-3	49423724	7.41	97.94	93.78	42.94
B-J-1	49001482	7.35	98.03	93.98	43.25
B-J-2	62342950	9.35	98.1	94.12	43.18
B-J-3	69382190	10.41	97.98	93.88	43.16
B-L-1	49811942	7.47	97.94	93.75	42.99
B-L-2	57342502	8.6	97.92	93.75	42.15
B-L-3	70559204	10.58	98	93.87	42.96
B-R-1	44100818	6.62	98.03	93.99	43.1
B-R-2	51350854	7.7	97.86	93.57	43.03
B-R-3	53366150	8	97.99	93.89	42.95

The functional annotations of all unigenes as annotated to the KEGG, NR, Swiss-Prot, Trembl, KOG, GO, and Pfam databases are presented in Supplementary Table 2. In *P. notoginseng*, 231,922 unigenes (100%) were annotated in the databases. A total of 144,866 (62.46%) transcripts exhibited significant matches in the NR database, whereas 115,980 (50.01%), 144,866 (62.46%), 103,136 (44.47%), 143,668 (61.95%), 90,888 (39.19%), 121,606 (52.43%), and 102,866 (44.35%) unigenes had significant matches in the KEGG, Swiss-Prot, Trembl, KOG, GO, and Pfam databases, respectively. The transcript sequences of *P. notoginseng* had 44.39% (*Daucus carota* subsp. sativus), 5.2% (*Vitis vinifera*), 3.37% (*Quercus suber*), 1.93% (*Olea europaea* var. sylvestris), 1.69% (*Coffea canephora*), and 1.35% (*Sesamum indicum*) similarity levels with related species (Supplementary Figure 1).

The GO terms were used to assign the unigene sets and classify the gene functions. In *P. notoginseng*, 121,606 transcripts were classified into three groups of GO terms, biological process (BP) (408,766), cellular component (161,751), and molecular function (365,692). These terms were further categorized into 59 subcategories. Cellular process, metabolic process and biological regulation were the most abundant terms in the BP category. The most dominant subcategories in the cellular component were cell, cell part and organelle. Binding and catalytic activity were the most represented terms in molecular function (Supplementary Figure 2). The annotated sequences were also mapped to the KEGG pathways. In *P. notoginseng*, 115,980 unigenes were assigned to 142 KEGG pathways. Notably, 1,597 sequences were annotated to the metabolism of terpenoids. These annotation results provided valuable information for analyzing metabolic pathways in *P. notoginseng*.

**FIGURE 1:**
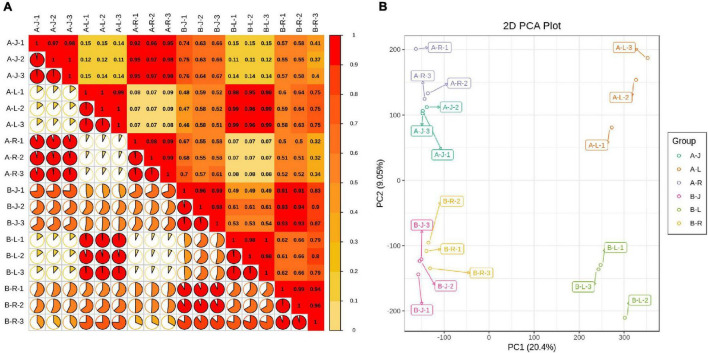
*Panax notoginseng* transcriptomic parameter analyses. **(A)** Pearson’s correlations between inflorescence-removal treatment and non-inflorescence-removal control samples; and **(B)** Principle component analysis of expressed genes.

Pearson’s correlations between the A-J and B-J samples ranged from 0.63 to 0.76 (Figure 1A). Pearson’s correlations between the A-L and B-L samples ranged from 0.95 to 0.99. Pearson’s correlations between the A-R and B-R samples ranged from 0.32 to 0.52. These results suggested that the inflorescence-removal treatment had the greatest effect on gene expression levels in the roots. The PCA showed that the A and B samples aggregated separately, indicating that significant differences existed in the gene expression profiles (Figure 1B).

### Differential Gene Expression Analysis of *P. notoginseng*

**FIGURE 2:**
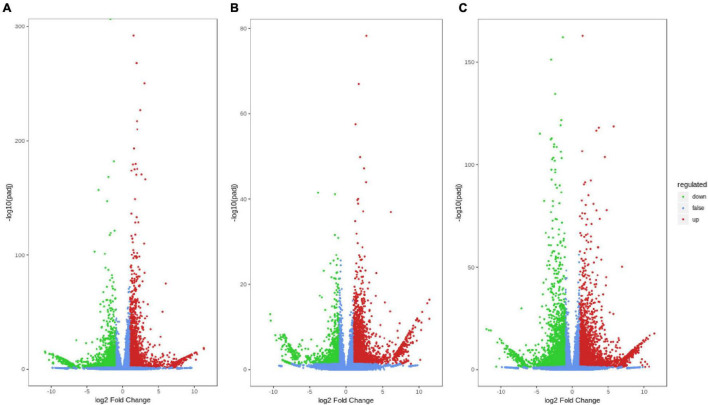
Differential gene volcano maps between inflorescence-removal treatment and non-inflorescence removal controls. **(A)** A-J vs. B-J **(B)** A-L vs. B-L **(C)** A-R vs. B-R. The significant DEGs with | Log_2_ (fold change)| ≥ 1 and FDR ≤ 0.05 are represented by green (down-regulated) and red (up-regulated) dots.

Three pairwise comparisons were established, A-L vs. B-L, A-J vs. B-J and A-R vs. B-R, to investigate the DEGs in *P. notoginseng* after an inflorescence-removal treatment. Volcano plots were constructed to determine the number of transcripts that were significantly changed after the inflorescence-removal treatment. The significant DEGs met the criteria | Log_2_ (fold change)| ≥ 1 and FDR ≤ 0.05. A total of 6,464 DEGs (2,996 up-regulated and 3,468 down-regulated) were identified in A-J vs. B-J, and 4,584 DEGs (3273 up-regulated and 1,311 down-regulated) were identified in A-L vs. B-L (Figures 2A,B). Additionally, 7,220 DEGs (3,815 up-regulated and 3,405 down-regulated) were found in A-R vs. B-R (Figure 2C).

The Venn diagram revealed that 14,374 DEGs were obtained, with 670 being shared among the three different organs (Supplementary Table 3). Moreover, 3,030, 4,462, and 3,658 DEGs were specifically expressed in the L, R, and J, respectively (Supplementary Figure 3). These results showed that the DEGs in the three *P. notoginseng* organs were differentially expressed.

**FIGURE 3:**
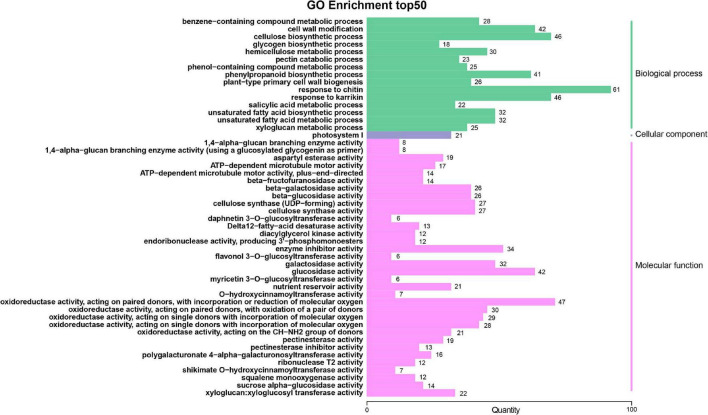
Gene ontology (GO) functional classifications of differentially expressed genes (DEGs) of A-J vs. B-J.

To identify the major functional terms enriched with DEGs, a GO enrichment analysis was carried out separately for three comparisons (A-J vs. B-J, A-L vs. B-L and A-R vs. B-R). For the GO enrichment, the DEGs of A-J vs. B-J, A-L vs. B-L and A-R vs. B-R were significantly enriched in 4,464, 3,827 and 4,435 GO terms, respectively, which belonged to three major functional categories: Cell Component (CC), Molecular Function (MF), and Biological Process (BP). The GO terms of A-J vs. B-J and A-R vs. B-R were similar (Figure 3 and Supplementary Figure 4). The most enriched components in the CC were categorized into photosystem I (GO: 0009522), whereas oxidoreductase activity (GO: 0016709) was the most-enriched term in the MF. For the BP category, the three mostly highly represented terms in the two pairwise comparisons were response to chitin (GO: 0010200), cellulose biosynthetic process (GO: 0030244) and response to karrikin (GO: 0080167). For the three pairwise comparisons, the top five terms in the BP category were response to chitin (GO: 0010200), response to karrikin (GO: 0080167), unsaturated fatty acid biosynthetic process (GO: 0006636), unsaturated fatty acid metabolic process (GO: 0033559) and cellulose biosynthetic process (GO: 0030244) (Figure 3 and Supplementary Figures 4, 5). Other terms related to energy metabolism, including cell wall modification (GO: 0042545), phenylpropanoid biosynthetic process (GO: 0009699), response to hydrogen peroxide (GO: 0042542), photosynthesis, light harvesting (GO: 0009765) and cellular transition metal ion homeostasis (GO: 0046916), were differentially enriched in the three pairwise comparisons.

**FIGURE 4:**
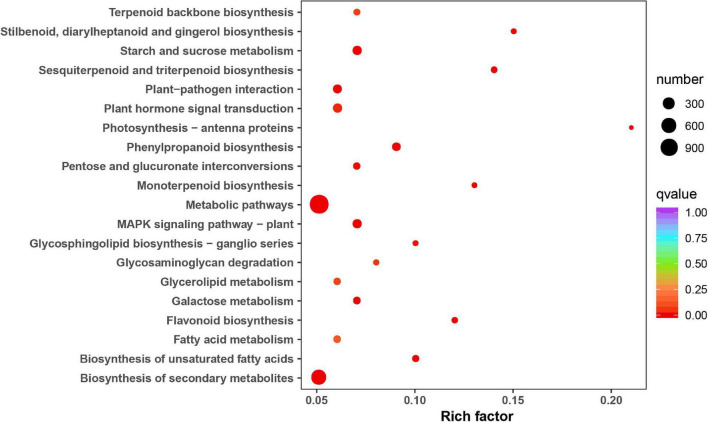
Statistics of KEGG enrichment of A-J vs. B-J.

The KEGG analysis revealed the key biological pathways involved in the responses to *P. notoginseng* inflorescence removal. Metabolic pathway, biosynthesis of secondary metabolites, plant hormone signal transduction, starch and sucrose metabolism and the MAPK signaling pathway were the top five significantly enriched pathways for A-J vs. B-J (Figure 4). The top five terms for A-L vs. B-L were metabolic pathway, biosynthesis of secondary metabolites, plant–pathogen interaction, fatty acid metabolism and the MAPK signaling pathway (Supplementary Figure 6). Metabolic pathway, biosynthesis of secondary metabolites, carbon metabolism, plant hormone signal transduction and starch and sucrose metabolism were the top five significantly enriched pathways for A-R vs. B-R (Supplementary Figure 7).

### Differentially Expressed Transcription Factor Genes After Inflorescence Removal

A total of 345 TFs were differentially expressed after inflorescence removal in A-J vs. B-J (Supplementary Table 4). These TFs were divided into 36 families. The major TFs identified in this study included WRKY (45), AP2/ERF (40), MYB (32), C2H2 (23), bHLH (22), NAC (18), and HB (18). Among these families, 41 WRKYs were up-regulated, while 4 WRKYs were down-regulated. In addition, 29 AP2s were up-regulated, while 11 AP2s were down-regulated. Furthermore, most of the TFs were up-regulated in A-J vs. B-J after inflorescence removal. For example, 17 and 6 C2H2s were up- and down-regulated, respectively, and 26 and 6 MYBs were up- and down-regulated, respectively.

In A-L vs. B-L, 217 TFs were differentially expressed after inflorescence removal (Supplementary Table 5). These TFs were divided into 26 families. The major TFs identified in this study included WRKY (52), AP2/ERF (25), MYB (23), bHLH (18), C2H2 (15), C2C2 (11), and Heat shock transcription factor (HSF) (11). Among these families, 48 and 4 WRKYs were up- and down-regulated, respectively, while 23 and 2 AP2s were up- and down-regulated, respectively. Furthermore, 22 MYBs were up-regulated, and 1 MYB was down-regulated.

In A-R vs. B-R, 398 TFs were differentially expressed after inflorescence removal (Supplementary Table 6). These TFs were divided into 39 families. The major TFs identified in this study included MYB (50), WRKY (36), AP2/ERF (39), bHLH (29), SBP (25), C2H2 (22), and NAC (21). Among these families, 24 and 26 MYBs were up- and down-regulated, respectively, while 32 and 4 WRKY s were up- and down-regulated, respectively. Furthermore, 22 AP2s were up-regulated, and 17 AP2s were down-regulated. Most of the TFs were up-regulated after inflorescence removal, indicating that these TFs mainly responded to the inflorescence-removal treatment through positive feedback regulation.

### Analysis of Genes Expression in the Saponin Terpenoid Backbone Biosynthesis Pathway of *P. notoginseng* in Response to Inflorescence Removal

**FIGURE 5:**
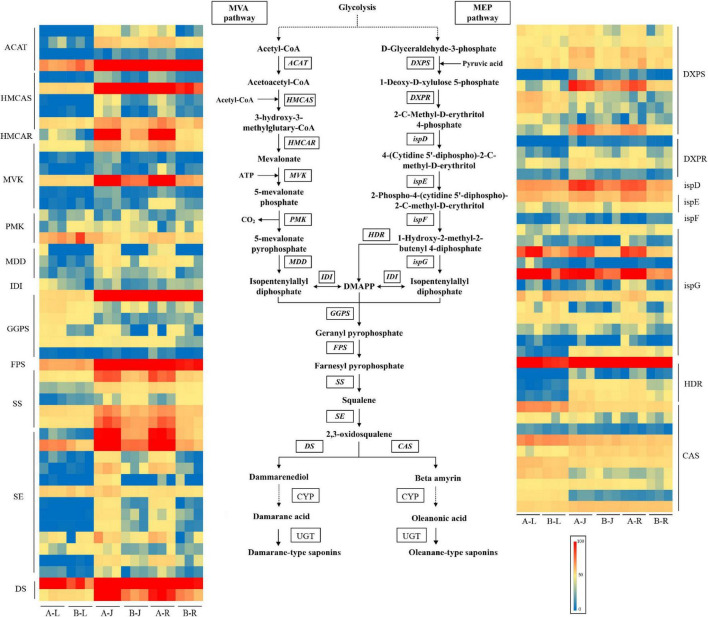
Expression levels of mevalonic acid pathway transcripts involved in saponin biosynthesis in *P. notoginseng.*

A total of 22 genes (245 transcripts) involved in saponin terpenoid backbone biosynthesis were identified through homology searches (Figure 5 and Supplementary Table 7). The annotation results showed that 20 genes (94 transcripts) encoding the enzymes involved in saponin biosynthesis in the MVA and MEP pathways were identified in *P. notoginseng*. Among them, 53 transcripts displayed differential expression levels in A-J vs. B-J, such as those encoding HMCAR, HMCAS, MVK, DXPS, SS, and SE. However, only 11 transcripts, representing eight gene families, were differentially expressed in A-L vs. B-L, such as those encoding DXPS, ispG, and GGPS. For A-R vs. B-R, 73 transcripts were differentially expressed after the inflorescence-removal treatment, such as those encoding ispG, DXPS, HMCAR, ACAT, MVK, HMCAS, SS, and SE. Notably, these identified DEGs were dramatically up-regulated after the inflorescence-removal treatment and were more highly expressed in root than in leaf.

In the MVA pathway of *P. notoginseng*, most of the genes were up-regulated after the inflorescence-removal treatment in the rhizome and root, including four ACAT transcripts (Cluster-10658.103649, Cluster-10658.117619, Cluster-10658.120546, and Cluster-10658.90144), five HMCAS transcripts (Cluster-10658.104575, Cluster-10658.121310, Cluster-10658.121476, Cluster-10658.91791, and Cluster-10658.91793), one HMCAR transcript (Cluster-10658.23096), six MVK transcripts (Cluster-10658.105056, Cluster-10658.124133, Cluster-10658.134107, Cluster-10658.172008, Cluster-10658.175434, and Cluster-10658.105057), and three Mevalonate diphosphosphate decarboxylase (MDD) transcripts (Cluster-10658.176645, Cluster-10658.92637, and Cluster-10658.28910). However, two Phosphomevalonate kinase (PMK) transcripts (Cluster-10658.129140 and Cluster-10658.137952) were down-regulated after inflorescence removal. In the MEP pathway of *P. notoginseng*, the expression levels of eight DXPS transcripts (Cluster-10658.100913, Cluster-10658.114918, Cluster-10658.120854, Cluster-10658.125787, Cluster-10658.77154, Cluster-10658.86726, Cluster-10658.122484, and Cluster-10658.126427), four DXPR transcripts (Cluster-10658.125260, Cluster-10658.89668, Cluster-10658.99642, and Cluster-10658.133602), one ispD transcript (Cluster-10658.52624), one ispE transcript (Cluster-10658.96587) and nine ispG transcripts (Cluster-10658.111637, Cluster-10658.126659, Cluster-10658.130396, Cluster-10658.111429, Cluster-10658.114749, Cluster-10658.115124, Cluster-10658.126921, Cluster-10658.61563, and Cluster-10658.89664) were high in the rhizome and root. However, one ispE transcript (Cluster-10658.75849) and one DXPS transcript (Cluster-10658.73494) were down-regulated in root after inflorescence removal. The effects of inflorescence removal on the expression levels of saponin terpenoid backbone biosynthesis-related genes in leaf were less than in other tissues. The most predominant expression level of one HDR transcript (Cluster-10658.100291) was observed in the root after inflorescence removal. Most of SS and SE transcripts were up-regulated after the inflorescence-removal treatment in the rhizome and root, including three SS transcripts (Cluster-10658.82094, Cluster-10658.118736, and Cluster-10658.116093) and nine SE transcripts (Cluster-10658.90339, Cluster-10658.56431, Cluster-10658.68954, Cluster-10658.37977, Cluster-10658.20921, Cluster-10658.26067, Cluster-10658.79097, Cluster-10658.37976, and Cluster-10658.79098). The two DS transcripts were highly expressed in the rhizome and root, but the expression changes of DS transcripts were not as large as SS and SE after the inflorescence-removal treatment. Like DS transcripts, the expression level of most CAS transcripts did not change significantly after the inflorescence-removal treatment.

**FIGURE 6:**
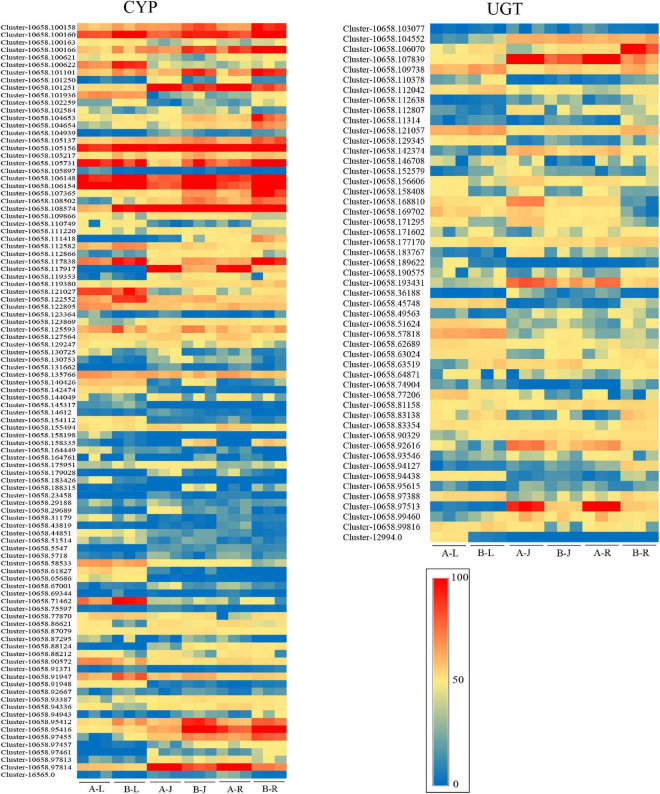
Expression levels of CYP and UGT transcripts in *P. notoginseng.*

The cytochrome P450-dependent monooxygenases (CYP) families had different expression profiles in the different tissues of *P. notoginseng*. A total of 100 CYP transcripts were identified in *P. notoginseng*, with 16, 13, 19, 7, 23, and 22 being most highly expressed in A-J, A-L, A-R, B-J, B-L, and B-R, respectively (Figure 6 and Supplementary Table 7). This indicated that the CYP transcripts were more highly expressed in the root (41%) than in the other two tissues of *P. notoginseng*. A total of 51 UGT transcripts were identified in *P. notoginseng*. Among these UGT transcripts, the expression levels of 10, 7, 7, 2, 17, and 8 were highest in A-J, A-L, A-R, B-J, B-L, and B-R, respectively (Figure 6 and Supplementary Table 7). These results indicated that the UGT transcripts were more highly expressed in the leaf (47.1%) than in the other two tissues of *P. notoginseng*.

### Metabolomics Responses of *P. notoginseng* to Inflorescence Removal

**FIGURE 7:**
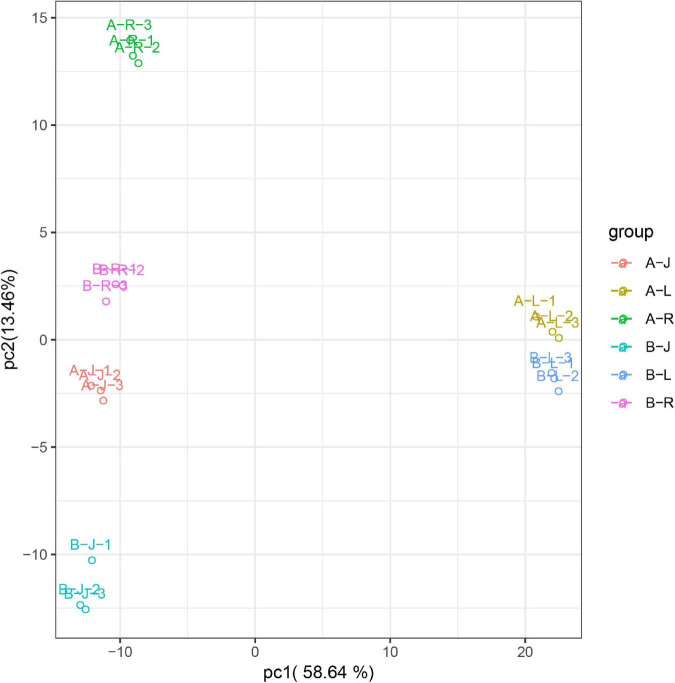
Metabolics analysis using a principal component analysis.

Before analyzing the DAMs, we performed a PCA to detect the degrees of variability between and within groups. As shown in Figure 7, the first two PCs could separate 18 samples clearly, accounting for 72.1% of the total variability. PC1 accounted for 58.64% of the variability, whereas PC2 accounted for 13.46% of the variability.

To further understand the changes in metabolism in *P. notoginseng* caused by inflorescence removal, an analysis of the widely targeted metabolome was performed using an LC-ESI-MS/MS system. Based on VIP ≥ 1.0 together with fold change ≥ 2 or ≤0.5 as thresholds for significant differences, the following 64 DAMs (46 up- and 18 down-regulated) were identified in A-J vs. B-J: 9 alkaloids, 9 amino acids and derivatives, 21 flavonoids, 4 lignans and coumarins, 2 nucleotides and derivatives, 2 organic acids, 4 phenolic acids, 1 quinone, 3 terpenoids and 9 other metabolites (Supplementary Table 8). In A-L vs. B-L, the following 42 DAMs (13 up- and 29 down-regulated) were identified: 4 alkaloids, 3 amino acids and derivatives, 9 flavonoids, 1 lignans and coumarins, 13 lipids, 1 nucleotides and derivatives, 1 organic acids, 2 phenolic acids, 5 terpenoids, and 3 other metabolites (Supplementary Table 9). In A-R vs. B-R, of the following 112 DAMs (92 up- and 20 down-regulated) were identified: 8 alkaloids, 19 amino acids and derivatives, 23 flavonoids, 6 lignans and coumarins, 6 lipids, 8 nucleotides and derivatives, 5 organic acids, 12 phenolic acids, 2 terpenoids, 1 tannin, and 22 other metabolites (Supplementary Table 10).

**FIGURE 8:**
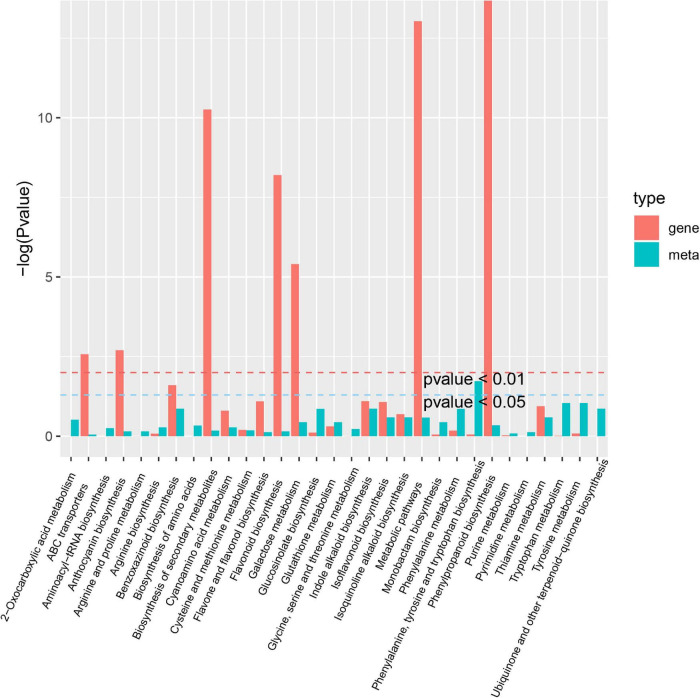
Joint KEGG enrichment *P*-value histogram of A-J vs. B-J.

A co-joint KEGG enrichment analysis was performed and showed that 16 of 64 DAMs were classified and assigned to 31 metabolic pathways in A-J vs. B-J. The main pathways were phenylalanine, tyrosine and tryptophan biosynthesis (ko00400), followed by tyrosine metabolism (ko00350) and tryptophan metabolism (ko00380) (Figure 8). Among 42 DAMs identified in A-L vs. B-L, 7 metabolites were allocated to 14 metabolic pathways. The most significantly enriched pathways were histidine metabolism (ko00340), lysine biosynthesis (ko00300) and tryptophan metabolism (ko00380) (Supplementary Figure 8). Of 112 DAMs in A-R vs. B-R, 38 metabolites were allocated to 52 metabolic pathways. The most significantly enriched pathways were 2-oxocarboxylic acid metabolism (ko01210), glucosinolate biosynthesis (ko00966) and ABC transporters (ko02010) (Supplementary Figure 9). Thus, the inflorescence-removal treatment mainly influenced the synthesis of amino acids and proteins and had the greatest effects on the metabolites in the roots of *P. notoginseng*.

**FIGURE 9:**
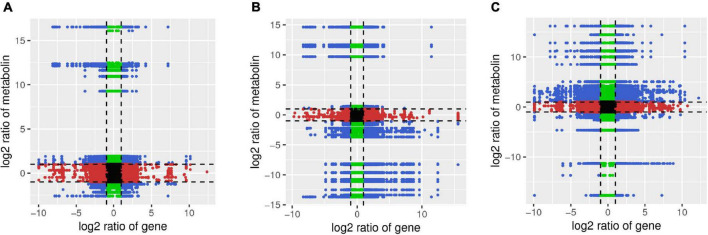
Quadrant diagram representing the association of transcriptomics and metabolomics variations in A-J vs. B-J **(A)**, A-L vs. B-L **(B)**, and A-R vs. B-R **(C)**. The black dotted lines represent the differential thresholds. Outside the threshold lines, there are significant differences in the gene/metabolites, whereas within the threshold lines the gene/metabolites are unchanged. Each point represents a gene/metabolite. Black dots = unchanged genes/metabolites, green dots = differentially accumulated metabolites with unchanged genes, red dots = differentially expressed genes with unchanged metabolites, and blue dots = both differentially expressed genes and differentially accumulated metabolites.

The Pearson’s correlation coefficients for nine quadrants are shown in Figure 9. The third and the seventh quadrants showed that the gene and metabolite differential expression patterns are consistent; genes are positively related to metabolite regulation and/or changes in metabolites may be positively regulated by genes. DEGs and DAMs having Pearson’s correlation coefficients greater than 0.8 were further selected and are presented as a heat map (Supplementary Figures 10–12).

### Integrated Analysis of Genes and Metabolites Related to *P. notoginseng* Ginsenoside Biosynthesis After Inflorescence Removal

It is estimated that there are more than 1,000 triterpenoid saponins. The structural diversity of these compounds is derived from various tailoring processes in the downstream pathway (Zhan et al., 2016). Triterpene saponins are derived from terpenoid backbone biosynthesis, followed by sesquiterpenoid and triterpenoid biosynthesis, which are then used as precursors by specific CYP450s and GTs for the formation of various ginsenosides (Rai et al., 2016). Combining transcriptomics with metabolomics data revealed that the inflorescence-removal treatment affected the late steps of ginsenoside biosynthesis.

**FIGURE 10:**
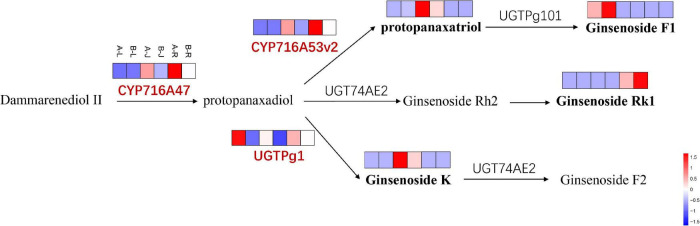
The DEGs and DAMs involved in ginsenoside biosynthesis in response to inflorescence removal.

The CYP716A47 and CYP716A53v2 genes encoding CYP450 enzyme catalyzed the formation of PPD from dammarenediol and protopanaxatriol from PPD, respectively (Li et al., 2017). In this study, the expression of CYP716A47 and CYP716A53v2 genes were significantly up-regulated in the root and rhizome of *P. notoginseng* but not significantly changed in the leaf of *P. notoginseng* after the inflorescence-removal treatment (Figure 10). The UGTPg1 gene was significantly up-regulated in the leaf, rhizome and root of *P. notoginseng*. The protopanaxatriol and ginsenoside K contents both showed significant up-regulation in the rhizome of *P. notoginseng* after the inflorescence-removal treatment, which consisted with expression of the two key genes. However, the ginsenoside F1 was down-regulated in the leaf of *P. notoginseng*. Ginsenoside Rk1 is a downstream product of protopanaxadiol, and its content decreased in the root of *P. notoginseng*.

### Integrated Analysis of Genes and Metabolites Related to *P. notoginseng* Flavonoid Biosynthesis After Inflorescence Removal

**FIGURE 11:**
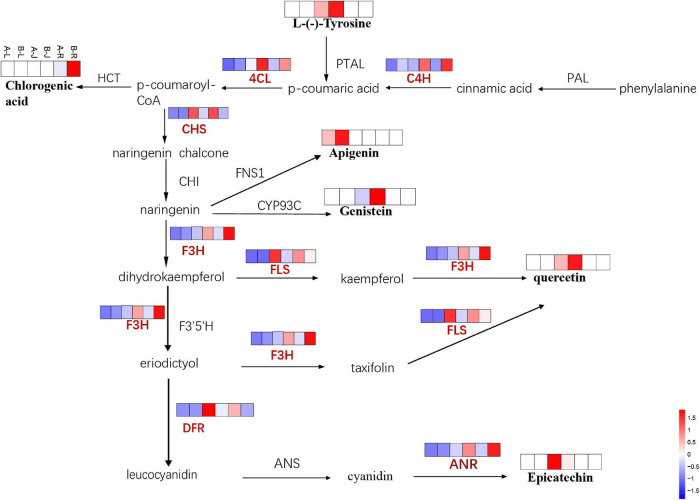
The DEGs and DAMs involved in flavonoid biosynthesis in response to inflorescence removal.

In addition to ginsenosides, flavonoid biosynthesis was influenced by the inflorescence-removal treatment. Combining transcriptomics with metabolomics data revealed that the inflorescence-removal treatment affected the entire flavonoid biosynthetic pathway (Figure 11). As a branch of phenylpropanoid biosynthesis, flavonoid biosynthesis was enriched with seven DEGs, four were down-regulated and three were up-regulated after the inflorescence-removal treatment. Two of the down-regulated genes (C4H and 4CL) are involved in the process of catalyzing cinnamic acid to form *p*-coumaroyl-CoA, whereas the other two down-regulated genes [F3H and anthocyanidin reductase (ANR)] directly affect flavone and flavonol biosynthesis and anthocyanin biosynthesis. The three up-regulated genes were chalcone synthase, flavonol synthase (FLS) and dihydroflavonol flavonol synthesis (DFR), which were also located upstream and downstream of the pathway.

Upstream of the pathway, the L-phenylalanine content was down-regulated significantly in A-J vs. B-J. Subsequently, chlorogenic acid is generated under the actions of C4H and 4CL, which showed significant down -regulation in the root of *P. notoginseng*. In addition, the apigenin and genistein contents were down-regulated in the leaf and rhizome of *P. notoginseng*, respectively. Afterward, naringenin produces dihydrokaempferol and eriodictyol under the actions of F3′5′H and F3H. The latter was significantly down-regulated in the rhizome and root of *P. notoginseng*, whereas the *FLS* gene was up-regulated in the rhizome and root of *P. notoginseng*. Quercetin is a downstream product of dihydrokaempferol and eriodictyol, and its content also decreased in the rhizome of *P. notoginseng*. The epicatechin content was significantly up-regulated, whereas the expression of ANR, which affects this process, was down-regulated in the rhizome of *P. notoginseng*.

## Discussion

Roots, stems, leaves, and flowers are the main organs of plants. These organs are not independent and damage to one organ can affect the growth and development of the plant (Wang et al., 2001). Flower removal changes the center of plant growth and material exchange, ultimately affecting plant yield and quality (Li et al., 2014). For example, tobacco (*Solanum tuberosum* L.) leaf yield and quality are significantly improved by apical dominance removal (Guo et al., 2002). The biological yield and total biomass of the organs of Jerusalem artichoke are increased by flower removal (Gao et al., 2020). Two-year field experiments showed that the primary root length and biomass accumulation, as well as the saikosaponin a and d contents, all increase under a continuous inflorescence-removal treatment (Wang et al., 2021). Although inflorescence removal or disbudding has been used to improve the yield and quality of rhizome crops, the underlying molecular mechanisms are unknown. Here, we compared the differences between the control and the inflorescence-removal treatment groups of *P. notoginseng* on transcriptomics and metabolomics levels.

To further study the effects of inflorescence removal on the metabolism of PNSs, the transcriptome and the expression levels of key enzymes in PNS metabolism were studied. The partial validation of DEG roles in inflorescence removal-related stress responses was obtained by performing GO and KEGG enrichment analyses. A KEGG enrichment is mainly used to analyze secondary metabolite biosynthesis. Thus, the effects of inflorescence removal on PNS synthesis in *P. notoginseng* root were assessed using this method. In the KEGG enrichment analysis, all 21 reported terpenoid backbone biosynthetic pathway genes were differentially expressed. For the MVA pathway, five DEGs in the root and rhizome of *P. notoginseng* were up-regulated after inflorescence removal. In A-L vs. B-L, most of the transcripts in both the MVA and MEP pathways did not show significant expression differences (Figure 5).

Plants produce a rich array of terpenoids with diverse structures, which play important roles in both basic biological processes and interactions with environmental stresses (Lamien-Meda et al., 2010). An inflorescence-removal treatment increases the PNS contents of the medicinal parts of *P. notoginseng* rhizomes and main roots (Liao et al., 2017). In this study, numerous genes involved in the MVA and MEP pathways were significantly up-regulated in the roots of *P. notoginseng* after the inflorescence-removal treatment. Among all the DEGs involved in PNS synthesis, HMCAR and DXPS are the rate-limiting enzymes of MVA and MEP pathways (Wang et al., 2021). HMCAR-overexpression cannot overcome the limitations of the MVA pathway, and additional, as yet unknown, mechanisms might regulate the MVA pathway and govern the pathway flow and subsequently metabolite yield (Liao et al., 2016). Consequently, we believe that the increase in the PNS content may be because MVKs in the MVA pathway were up-regulated, which could increase isopentenyl diphosphate synthesis (Mao et al., 2014). Additionally, the key gene of terpenoid backbone biosynthesis, GGPS, was up-regulated (Figure 5). GGPS controls geranyl diphosphate synthesis, which precedes sesquiterpenoid and triterpenoid biosynthesis (saponin backbone biosynthetic pathway) (Pribat et al., 2013). The higher GGPS transcript levels caused an increase in terpenoid precursor yields and consequently influenced the downstream synthesis of terpenoids. This may be a reason for the PNS increase in *P. notoginseng* roots after inflorescence-removal treatment. Additionally, the high DXPS expression level mainly increases the contents of other terpenoids, such as diterpene, rather than saikosaponin and triterpenoids. Moreover, DXPS showed a higher expression level in leaves than in roots of several medicinal plants (Sharma et al., 2016). This indicated that DXPS contributed more to the synthesis of other terpenoids compared with saikosaponin and triterpenoids. To enhance triterpene saponins productions, key enzyme genes PnSS and PnHMGR were introduced into *P. notoginseng* cells and the results showed that overexpression of SS could remarkably enhance the accumulation of total saponins (Deng et al., 2017). SE is highly expressed in root compared with stem and leaf (He et al., 2008). In this study, the expression level of SE in root was similar to that in rhizome. Some studies have shown that overexpression of DS gene can increase the content of saponins in *P. notoginseng* (Zheng et al., 2021). The inhibition of CAS expression can decrease the synthesis metabolic flux of the phytosterol branch and promote the accumulation of *P. notoginseng* saponins (Yang et al., 2017). In this study, the expression of DS and CAS was not significantly affected by the inflorescence-removal treatment.

Different parts of *P. notoginseng* undergo varied levels of triterpene saponin synthesis. Roots are rich in protopanaxatriol- and PPD type saponins, whereas leaves and flowers contain PPD -type saponins only (Wan et al., 2006). The putative candidate genes involved in triterpene saponin biosynthesis may be mainly CYP450s and GTs, which could account for the synthesis and accumulation of triterpene saponins in specific organs (Yendo et al., 2010). UGTPg45 selectively transfers a glucose moiety to the C3 hydroxyl of PPD to form ginsenoside Rh2, whereas UGTPg29 selectively transfers a glucose moiety to the C3 hydroxyl Rh2 to form ginsenoside Rg3 (Wang et al., 2015). CYP716A94 is a b-amyrin 28-oxidase involved in oleanolic acid production from b-amyrin, and CYP72A397 is an oleanolic acid 23-hydroxylase involved in hederagenin production from oleanolic acid (Han et al., 2018). PgUGT74AE2 catalyzes the transfer of a glucose moiety from UDP-glucose to the C3 hydroxyl groups of PPD and compound K, yielding Rh2 and F2, respectively, and PgUGT94Q2 transfers a glucose moiety from UDP-glucose to Rh2 and F2 to generate Rg3 and Rd, respectively (Jung et al., 2014). Thus, CYP and UGT transcripts are involved in saponin synthesis. In this study, 100 and 51 members of the CYP450 and UGT gene families, respectively, were identified (Supplementary Table 7).

Transcription factors are major regulators of plant development and responses to external stimuli, and those in the AP2/ERF, bHLH, MYB, NAC, and WRKY gene families are involved in plant biotic and abiotic stress responses (Seo and Choi, 2015). Here, the WRKY family had the largest number of DEGs after inflorescence removal, indicating that members of this family play integral roles in *P. notoginseng*’s response to inflorescence removal. The nucleotide sequence of McWRKY75 contains a W-box motif that interacts with the promoters of other TF genes to regulate their transcriptional levels. Thus, McWRKY75 appears to act as a signaling molecule to activate other regulatory factors, or it interacts with other proteins (Eulgem et al., 2000). In addition, WRKY TFs are involved in the regulation of secondary metabolism, including the synthesis of anthocyanins and flavonoids (Pellegrini et al., 2018). One of the larger groups of plant TFs is the MYB protein family, and the members play regulatory roles in developmental processes and defense responses (Fornara and Coupland, 2009). In our TF analysis, a large number of flavonoid-related differentially expressed TFs were identified in the roots of *P. notoginseng* after the inflorescence-removal treatment. MYB and MYB-related TFs displayed the highest induction levels by the inflorescence-removal treatment. As reported, MYB75 promotes anthocyanin accumulation and volatile aroma production, including terpene volatiles, in tomato fruit (Jian et al., 2019). These results indicate possible links between the flavonoid pathway and terpene accumulation.

CYP450s are known to catalyze the oxidation function of carbon–carbon bonds as well as alkyl hydroxylation and hydroxyl oxidation reactions (Guo et al., 2014). The CYP enzyme (CYP716A47) was identified to be involved in the hydroxylation of dammarenediol-II at the C-12 position to yield PPD (Han et al., 2011). After that, two additional CYP716A subfamily genes (CYP716A52v2 and CYP716A53v2) were isolated and the gene product of CYP716A53v2 is a PPD 6-hydroxylase that catalyzes the formation of protopanaxatriol from PPD. Both CYP716A47 and CYP716A53v2 mRNAs accumulated ubiquitously in all organs of ginseng plants (Han et al., 2012). In general, glycosylation is the last step in the biosynthesis of secondary metabolites. Recently, identified eight GTs (UGT73C11, UGT73C10, UGT74AE2, UGT94Q2, UGT71A27, UGTPg1, UGTPg100, and UGTPg101) involved in the later steps of ginsenoside biosynthesis in the closely related species *P. ginseng* (Yan et al., 2014). UGTPg1 specifically glucosylates the C-20S-OH of dammarane-type triterpenoids, and catalyzes the conversion of PPD to CK (Yan et al., 2014). PgUGT74AE2 transfers a glucose moiety from UDP-glucose (UDP-Glc) to the C3 hydroxyl groups of PPD and compound K to form Rh2 and F2, respectively (Jung et al., 2014). It has been demonstrated that UGTPg101 catalyzes PPT to produce F1, followed by the generation of ginsenoside Rg1 from F1 (Wei W. et al., 2015). In this study, the expression levels of CYP716A47, CYP716A53v2, and UGTPg1 genes were up-regulated compared with untreated group, which consisted with production of protopanaxatriol and ginsenoside K in the rhizome of *P. notoginseng*. The results demonstrated a positive association between secondary metabolite content and expression of key gene in secondary metabolism pathway.

Flavonoids act as important indicators of *P. notoginseng* quality. Flavonoids are a group of polyphenol compounds with known antioxidant activities, and they are composed of flavones, flavonols, flavanones, anthocyanins and isoflavones (Nijveldt et al., 2001). Removing the inflorescence stem triggers events in *Arabidopsis*, including pigment accumulation and changes in gene expression of a subset of stress-induced genes, in a tissue distant from the wound site (Li and Strid, 2005). In this study, we found that the decrease in flavonoid accumulation was accompanied by changes in gene expression in *P. notoginseng*’s response to inflorescence removal. Among the various DEGs, the expression levels of C4H, 4CL, F3H, and DFR were positively correlated with the flavonoid concentration, whereas chalcone synthase, FLS and ANR were negatively correlated. Apigenin, genistein and quercetin were down-regulated 2. 17-, 3, 172-, and 2.06-fold after inflorescence removal (Supplementary Table 10). C4H, 4CL, and DFR are the key branch-point genes that regulate flavonoid accumulation (Liu et al., 2020). Expression of CHS gene was up-regulated and anthocyanin accumulation occurs in rosette leaves of *Arabidopsis* after removal of the inflorescence stem (Li and Strid, 2005). Our studies showed that low C4H and 4CL expression levels may be negatively correlated to flavonoid accumulation in *P. notoginseng*. The main physiological functions of quercetin and kaempferol in plants are scavenging reactive oxygen species and detoxifying free radicals, which increase plant tolerance to environmental changes (Belinha et al., 2007). However, information on flavonoids in inflorescence removal-treated plants is limited. Both F3H and F3′5′H play key roles in affecting the composition of dihydroxylated and trihydroxylated flavonoids (Wei K. et al., 2015). In this study, quercetin decreased during inflorescence removal, and the corresponding gene, F3H, was down-regulated in the rhizomes of *P. notoginseng*. This suggested that the decrease in quercetin was closely linked with F3H expression. Additionally, we noticed that epicatechin increased nearly fourfold after inflorescence removal and speculated that epicatechin was regulated by the DFR gene in *P. notoginseng*.

Overall, inflorescence-removal treatment is pivotal for the improvement of PNS and the regulation of flavonoid biosynthesis in *P. notoginseng*. However, whether inflorescence removal-induced wounding signals or the inflorescence removal-induced abolishment of reproductive growth has a strong regulatory effect on PNS biosynthesis remains unclear. Further investigations are underway. In addition, an integrated analysis of metabolomes and transcriptomes could reveal the correlated genes regulating the accumulations of active compounds and provide useful information for understanding the underlying biosynthesis-related molecular mechanisms. Our study offers useful data for investigating the molecular and chemical components of saponin biosynthesis in *Panax* plants.

## Data Availability Statement

The datasets presented in this study can be found in online repositories. The names of the repository/repositories and accession number(s) can be found below: NCBI SRA BioProject, accession no: PRJNA758433.

## Author Contributions

ZS and ZZ participated in research design. HL, JP, SZ, and YG conducted the experiments. YB and YX performed data analysis. YB and ZZ wrote or contributed to the writing of the manuscript. All authors contributed to the article and approved the submitted version.

## Conflict of Interest

The authors declare that the research was conducted in the absence of any commercial or financial relationships that could be construed as a potential conflict of interest.

## Publisher’s Note

All claims expressed in this article are solely those of the authors and do not necessarily represent those of their affiliated organizations, or those of the publisher, the editors and the reviewers. Any product that may be evaluated in this article, or claim that may be made by its manufacturer, is not guaranteed or endorsed by the publisher.

## Footnotes

1
www.metware.cn

2
https://code.google.com/p/corset-project/

## Abbreviations

PNS: *Panax notoginseng* saponin
MVA: mevalonic acid
MEP: methylerythritol phosphate
PPD: protopanaxadiol
KEGG: Kyoto encyclopedia of genes and genomes
NR: non-redundant
COG: Clusters of Orthologous Groups
KOG: euKaryotic Ortholog Groups
GO: gene ontology
DEG: differentially expressed gene
VIP: variable importance of the projection
PCA: principal component analysis
DAM: differentially accumulated metabolite
CC: cell component
MF: molecular function
BP: biological process
FPKM: fragments per kilobase of transcript per million fragments mapped
FDR: false discovery rate
LIT: linear ion trap
LC-ESI-MS/MS: liquid chromatography-electrospray ionization-tandem mass spectrometry
TFs: transcription factors
HSF: heat shock transcription factor
HMCAR: 3-hydroxy-3-methylglutaryl coenzyme-A reductase
HMCAS: 3-hydroxy-3-methylglutaryl coenzyme-A synthase
MVK: mevalonate kinase
DXPS: 1-deoxy-D-xylulose-5-phosphate synthase
ispG: 4-hydroxy-3-methylbut-2-enyl-diphosphate synthase
GGPS: geranylgeranyl pyrophosphate synthase
ACAT: acetyl-CoA acetyltransferase
MDD: mevalonate diphosphosphate decarboxylase
PMK: phosphomevalonate kinase
DXPR: 1-deoxy-D-xylulose-5-phosphate reductoisomerase
ispD: 2-*C*-methyl-D-erythritol 4-phosphate cytidylyltransferase
ispE: 4-diphosphocytidyl-2-*C*-methyl-D-erythritol kinase
HDR: 4-hydroxy-3-methylbut-2-enyl diphosphate reductase
CYP: cytochrome P450-dependent monooxygenases
UGT: UDP glycosyltransferases
PAL: phenylalanine ammonia-lyase
C4H: *Trans*-cinnamate 4-monooxygenase
4CL: 4-coumarate–CoA ligase
F3H: flavanone 3- hydroxylase
ANS: anthocyanidin synthase
ANR: anthocyanidin reductase
FLS: flavonol synthase
DFR: dihydroflavonol flavonol synthesis
F3′5′H: flavonoid 3′,5′-hydroxylase.
