# Supplementary material for: Transcriptomics and Metabolomics Changes Triggered by Inflorescence Removal in Panax notoginseng (Burk.)
Source: Front Plant Sci. 2021 Nov 12;12:761821. doi: 10.3389/fpls.2021.761821 (PMC8636121; doi:10.3389/fpls.2021.761821)
Supplement: Supplementary file 1 [file Data_Sheet_1.docx]

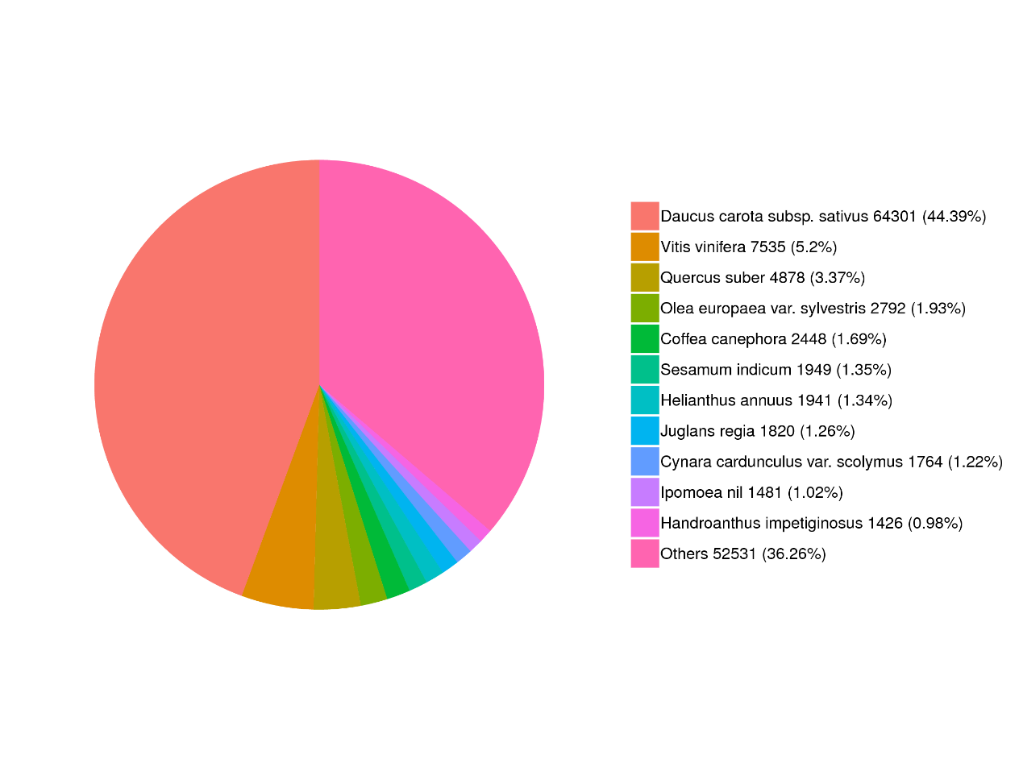


Fig. S1 Species distribution of the top BLAST hits of *P. notoginseng* unigenes when the encoded proteins were queried against the NCBI nonredundant protein database.


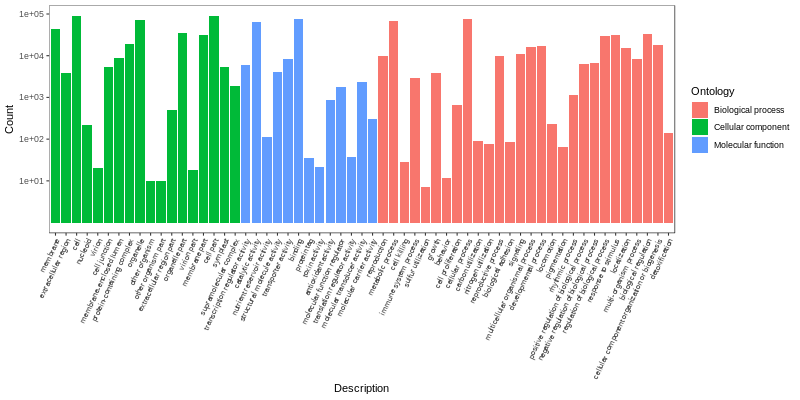


Fig. S2 Gene ontology classification of the *P. notoginseng* unigenes


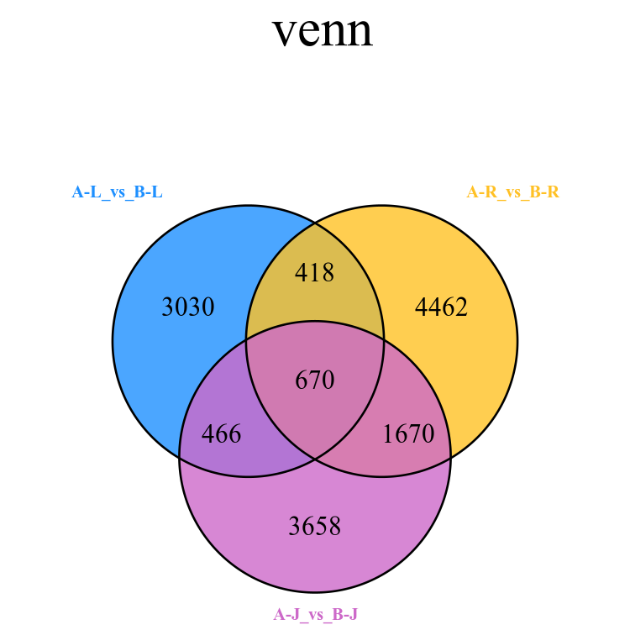


Fig. S3 Venn diagram of differentially expressed genes (DEGs).


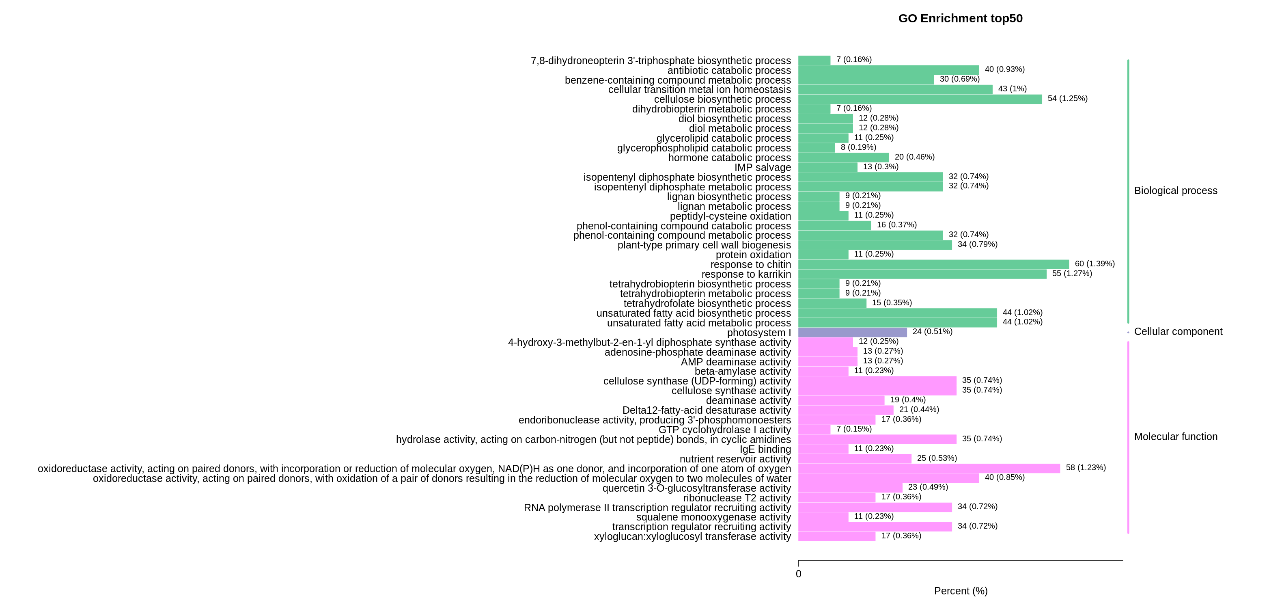


Fig. S4 Gene ontology (GO) functional classifications of differentially expressed genes (DEGs) of A-R vs. B-R


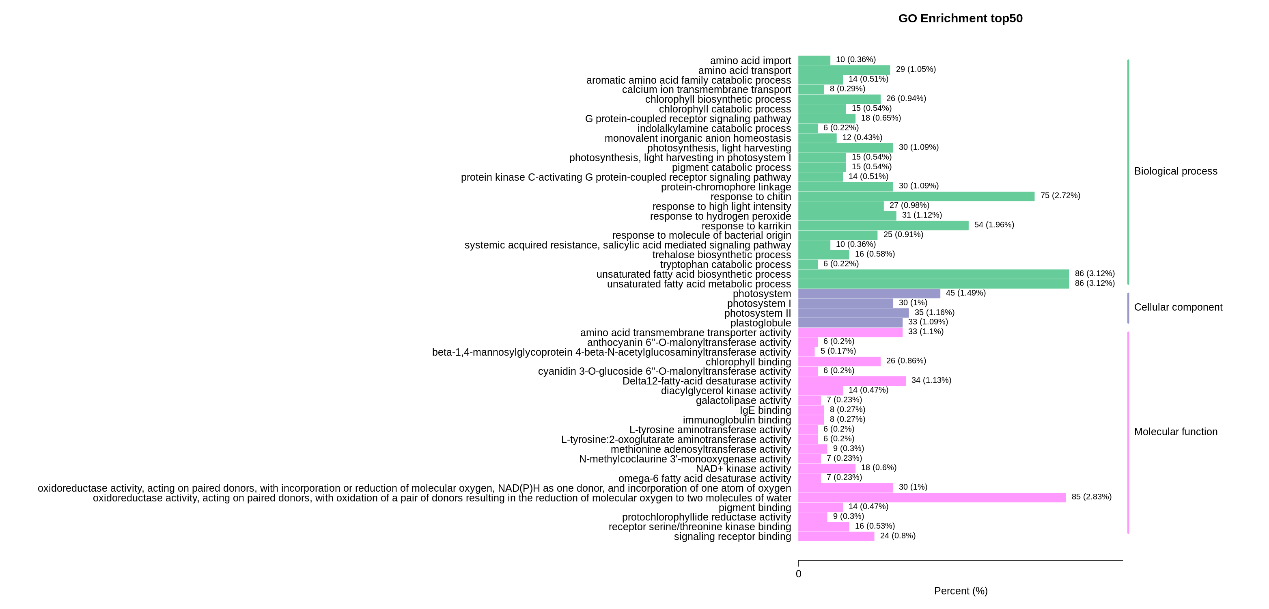


Fig. S5 Gene ontology (GO) functional classifications of differentially expressed genes (DEGs) of A-L vs. B-L


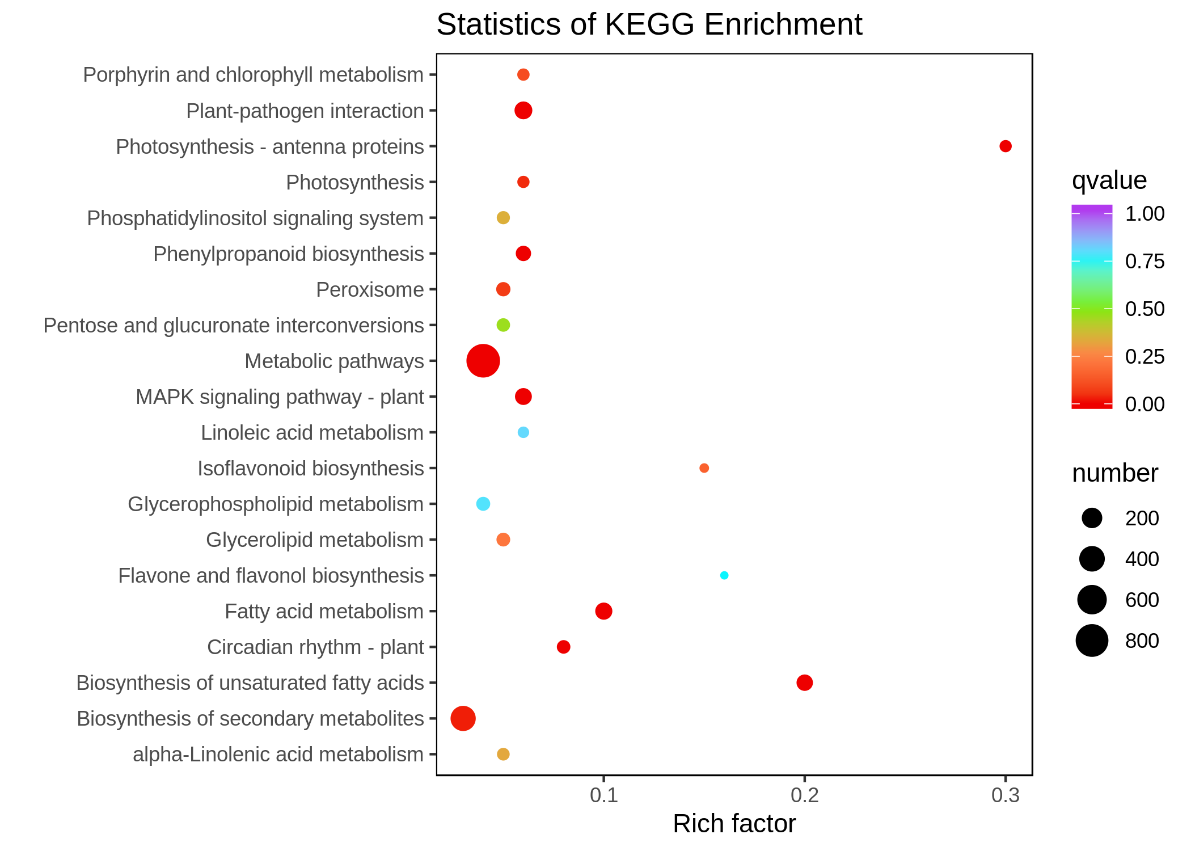


Fig. S6 Statistics of KEGG enrichment of A-L vs. B-L


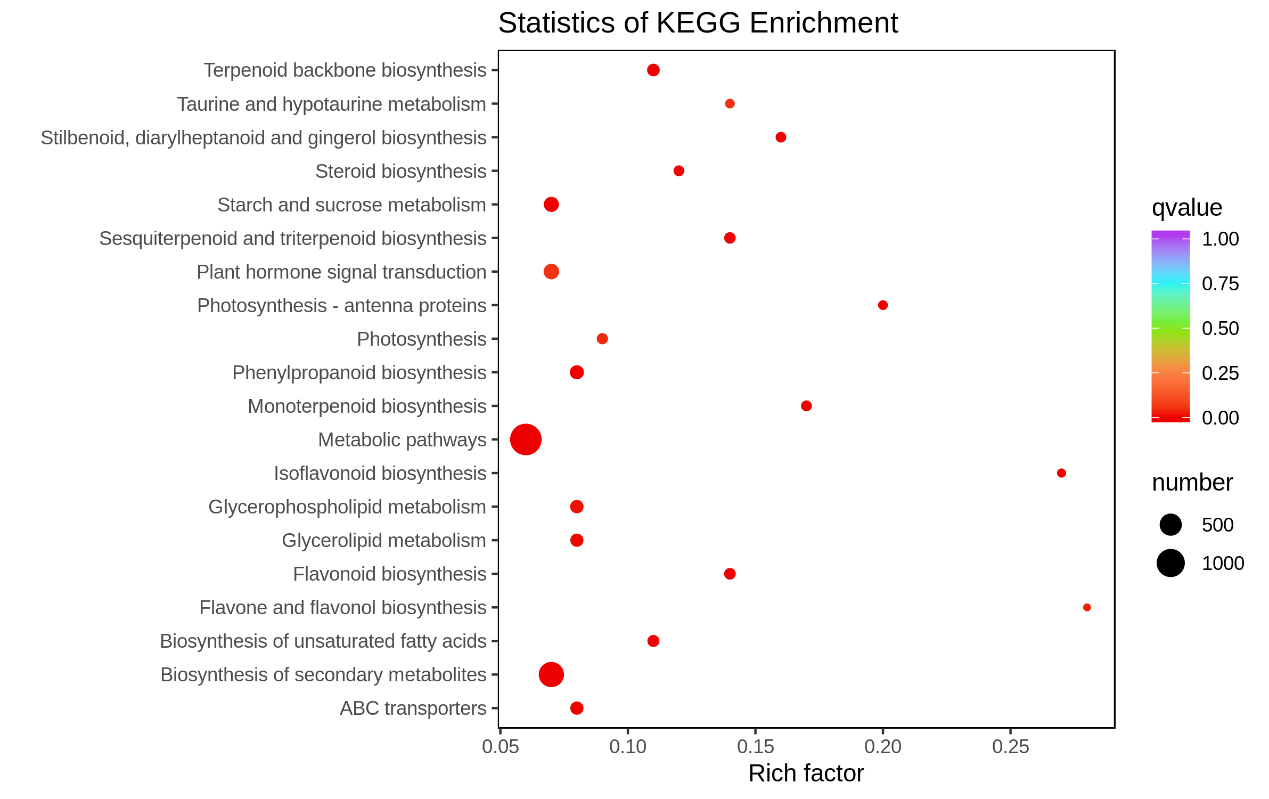


Fig. S7 Statistics of KEGG enrichment of A-R vs. B-R


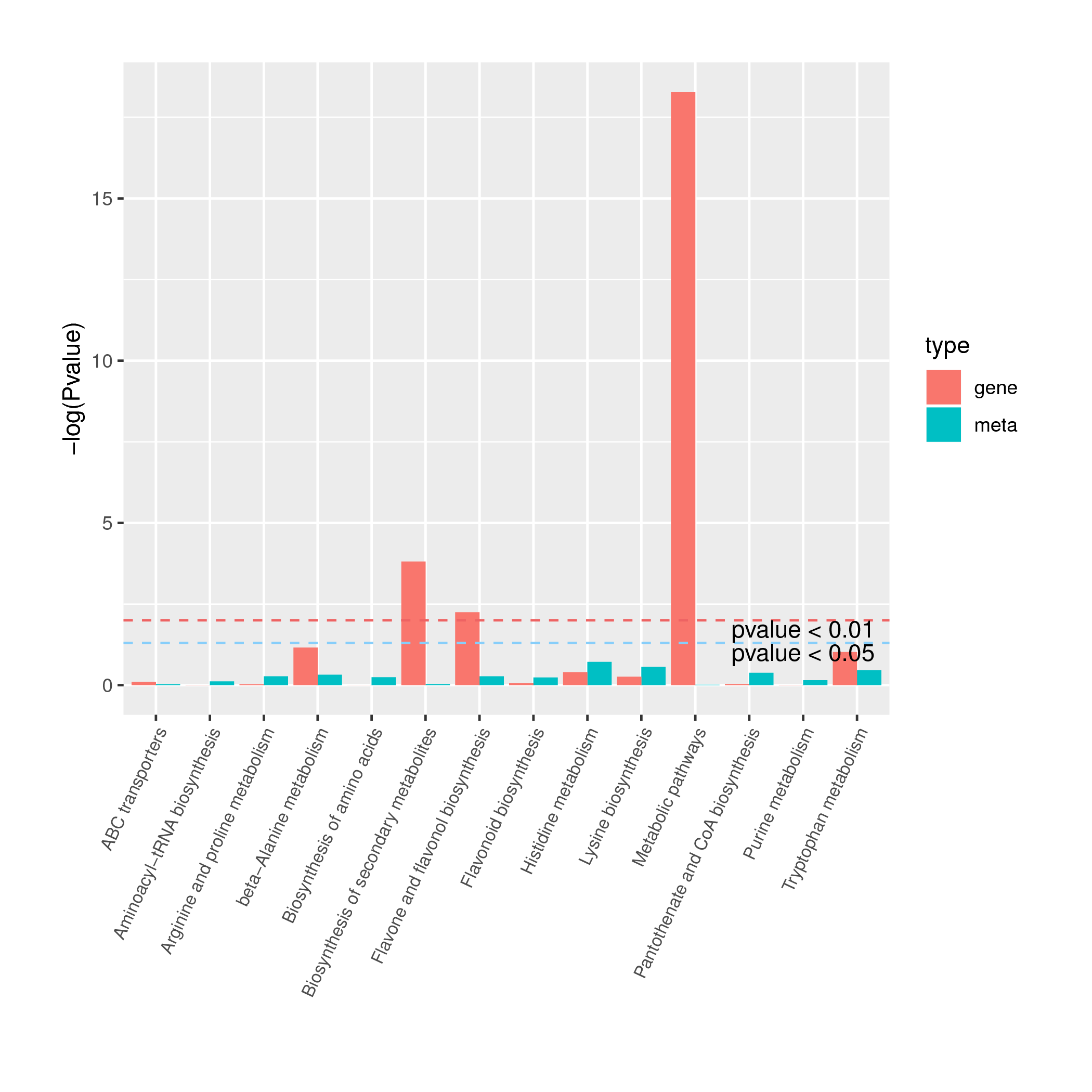


Fig. S8 Joint KEGG enrichment p-value histogram of A-L vs. B-L


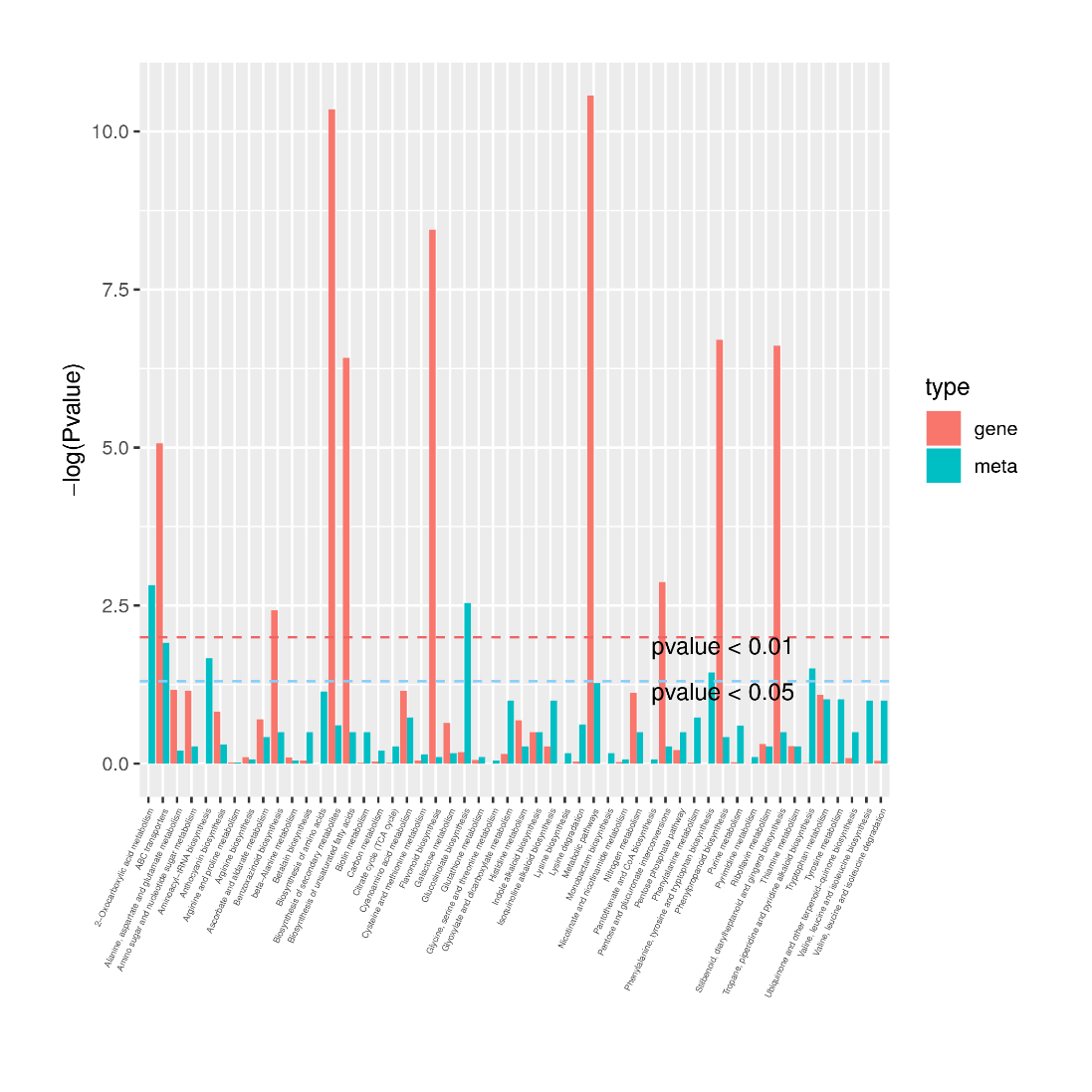


Fig. S9 Joint KEGG enrichment p-value histogram of A-R vs. B-R


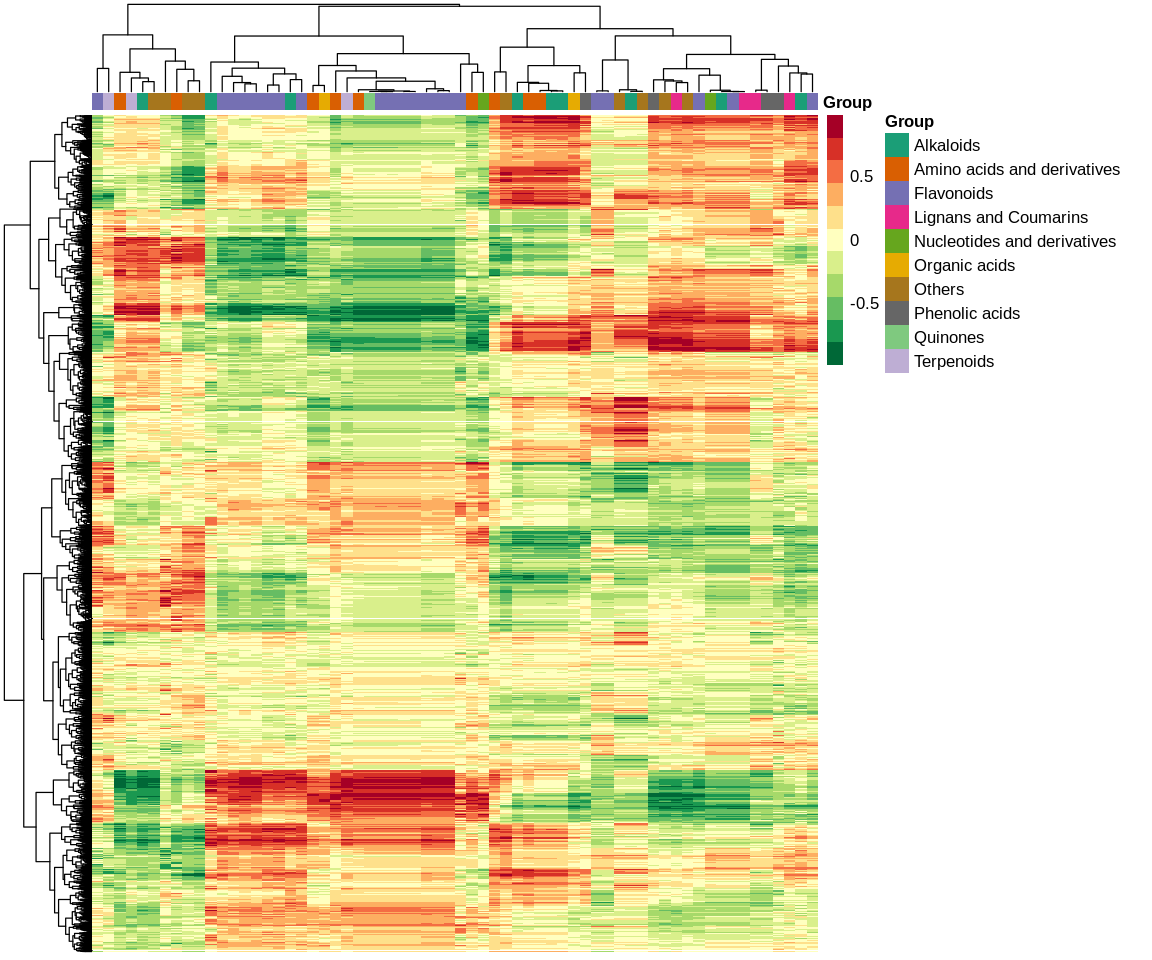


Fig. S10 Correlation coefficient cluster heat map of A-J vs. B-J


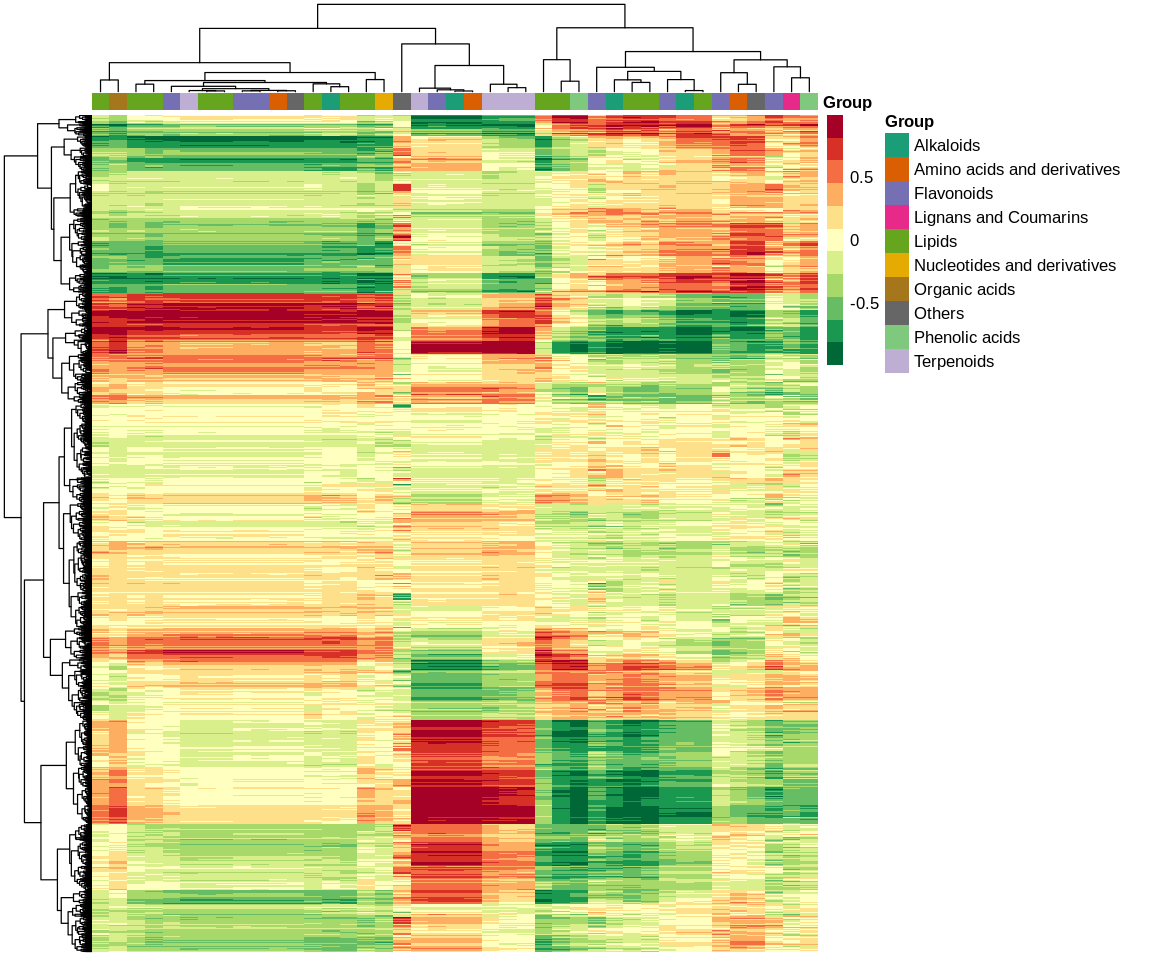


Fig. S11 Correlation coefficient cluster heat map of A-L vs. B-L


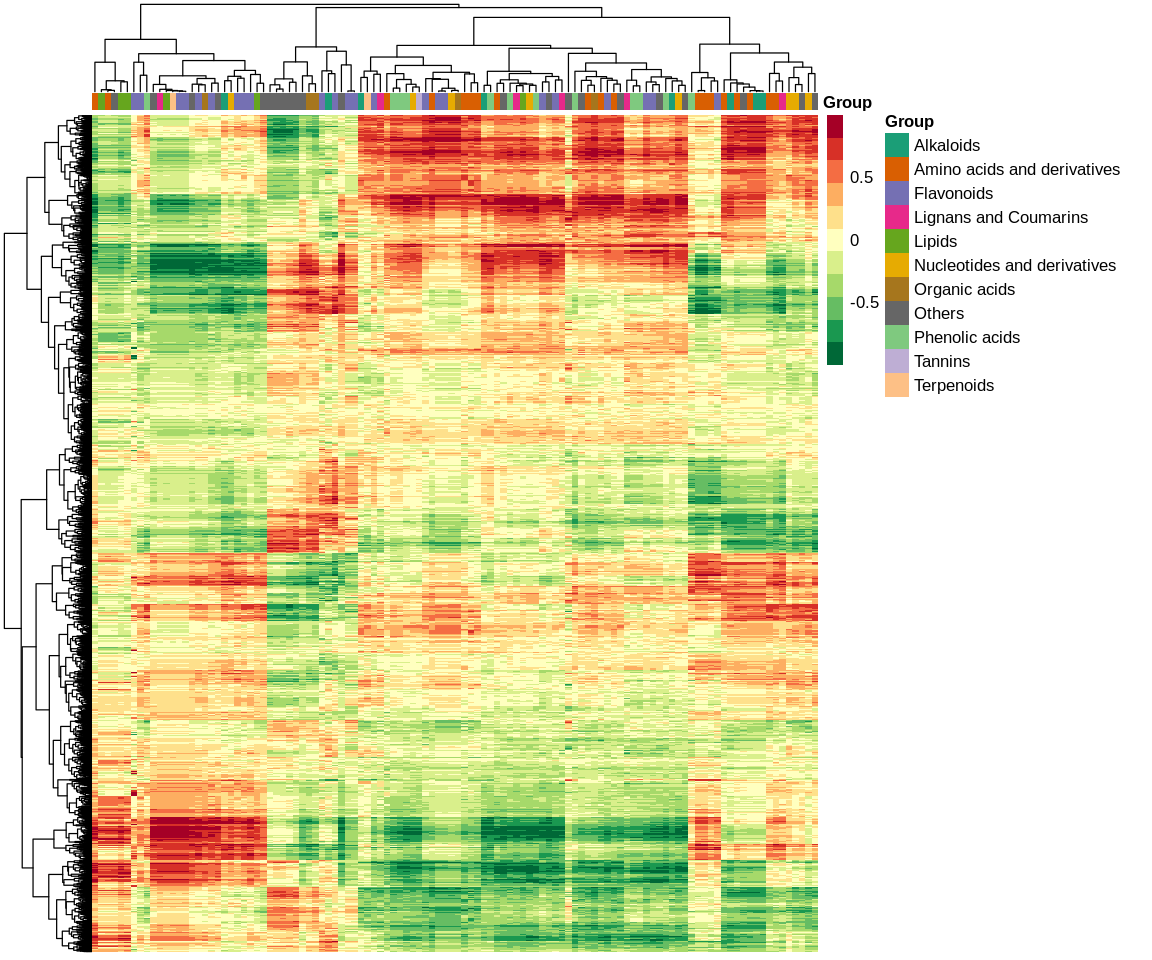


Fig. S12 Correlation coefficient cluster heat map of A-R vs. B-R
